# 

**Published:** 1897-05

**Authors:** 


### South Texas Medical Association.

The second annual meeting will be held in Galveston on Friday, May 14th, inst., at the Tremont hotel. All reputable physicians are invited to attend. The following papers will be read:

1. Ligation of the Common Carotid—Dr. R. T. Morris, Houston.

2. Operative Interference in the Removal of Tumors of the Upper Cervial Region and Face—Dr. J. E. Thompson, Galveston.

3. Etiology and Treatment of Dysentery—Dr. H. A. West, Galveston.

4. Anatomy of the Kidney—Dr. Bat Smith, Wharton.

5. Lipomata—Dr. Sofie Herzog, Brazoria.

6. Wound Grafting with Tissues from the Lower Animals—Dr. C. W. Trueheart, Galveston.

7. Consideration of Catarrhal Deafness in the Young—Dr. J. A. Mullen, Houston.

8. The Management of Abortion—Dr. R. W. Knox, Houston.

9. Contra Indications to the Use of the Coal-Tar Derivatives—Dr. B. F. Calhoun, Beaumont.

10. Subject unannounced—Dr. E. P. Daviss, Houston.

11. Subject unannounced—Dr. David Cerna, Galveston.

12. Some Practical Observations in Pelvic Operations—Dr. J. H. Sampson, Houston.

13. The Phonendoscope and its Use—Dr. O. L. Norseworthy, Houston.

E. S. Ferguson, M. D., Secretary,
Houston, Texas.

Bat Smith, M. D., President,
Wharton, Texas.

---

### The Fiftieth Anniversary of the American Medical Association.—Annual Announcement.

Office of the Permanent Secretary,
1400 Pine St., Philadelphia.

The Forty-eighth* Annual Session will be held in Philadelphia, Pa., on Tuesday, Wednesday, Thursday and Friday, June 1, 2, 3 and 4, commencing on Tuesday, at 10 a. m.

"The delegates shall receive their appointment from permanently organized State medical societies, and such county and district medical societies *as are recognized by representation in their respective State societies*, and from the medical departments of the Army and Navy and the Marine-Hospital service of the United States.

---

*There were no meetings held by the Association during the years 1861 and 1862.

"Each State, county and district medical society entitled to representation shall have the privilege of sending to the Association one delegate for every ten of its regular resident members, and one for every additional fraction of more than half that number; provided, however, that the number of delegates for any particular State, Territory, county, city or town shall not exceed the ratio of one in ten of the resident physicians who may have signed the Code of Ethics of the Association."

*Members by Application.*—Members by application shall consist of such members of the State, county and district medical societies entitled to representation in this Association as shall make application in writing to the Treasurer, and accompany said application with a certificate of good standing, signed by the president and secretary of the society of which they are members, and the amount of the annual subscription fee, $5. They shall have their names upon the roll, and have all the rights and privileges accorded to *permanent members*, and shall retain their membership upon the same terms.

The following resolution was adopted at the session of 1888: *That in future each delegate or permanent member shall, when he registers, also record the name of the Section, if any, that he will attend, and in which he will cast his vote for Section officers.*

Secretaries of medical societies, *as above designated*, are earnestly requested to forward, *at once*, lists of their delegates.

ADDRESSES.

The Presidential Address..................Nicholas Senn, Chicago.
Address in Surgery....................Wm. W. Keen, Philadelphia.
Address in Medicine........................Austin Flint, New York.
Address in State Medicine............John B. Hamilton, Chicago.

COMMITTEE OF ARRANGEMENTS.

H. A. Hare, 222 S. 15th Street, Philadelphia.

